# Relationship between Feline Knee Joint Osteoarthritis and Bone Mineral Density Quantified Using Computed Tomography and Computed Digital Absorptiometry

**DOI:** 10.3390/ani14172615

**Published:** 2024-09-09

**Authors:** Joanna Bonecka, Bernard Turek, Krzysztof Jankowski, Marta Borowska, Tomasz Jasiński, Graham Smyth, Małgorzata Domino

**Affiliations:** 1 Department of Small Animal Diseases and Clinic, Institute of Veterinary Medicine, Warsaw University of Life Sciences (WULS–SGGW), 02-787 Warsaw, Poland; joanna_bonecka@sggw.edu.pl; 2 Department of Large Animal Diseases and Clinic, Institute of Veterinary Medicine, Warsaw University of Life Sciences (WULS–SGGW), 02-787 Warsaw, Poland; bernard_turek@sggw.edu.pl (B.T.); tomasz_jasinski@sggw.edu.pl (T.J.); 3 Institute of Mechanics and Printing, Warsaw University of Technology, 02-524 Warsaw, Poland; krzysztof.jankowski1@pw.edu.pl; 4 Institute of Biomedical Engineering, Faculty of Mechanical Engineering, Białystok University of Technology, 15-351 Bialystok, Poland; m.borowska@pb.edu.pl; 5 Menzies Health Institute Queensland, Griffith University School of Medicine, Southport, QLD 4222, Australia; grahamcsmyth@gmail.com

**Keywords:** degenerative joint disease, stifle joint, radiography, density standard, cat

## Abstract

**Simple Summary:**

Osteoarthritis (OA) is a common and painful condition affecting 91% of cats, with knee joint OA present in 50% of cases. Diagnosing feline knee joint OA typically involves clinical examination and radiographic imaging, which also provides information about bone health, including bone mineral density (BMD). While the link between knee joint OA and BMD is well-established in humans, it has not been studied in cats. Understanding this relationship could shed light on how bone changes contribute to OA. This study aims to measure the BMD of cat knee joints, compare different BMD measurement methods, and explore how BMD changes with various severities of OA. For this purpose, 46 cat knee joints were analyzed using two techniques: computed tomography (CT) for the volumetric BMD (vBMD) and conventional radiography with a method called computed digital absorptiometry (CDA) for relative BMD (rBMD). This study found a positive correlation between vBMD and rBMD in key areas of the knee, such as the distal femur, patella, and proximal tibia. Additionally, this study adjusted rBMD for bone size differences (corrected rBMD) because variations in bone width were observed. All measures—vBMD, rBMD, and corrected rBMD—differed between normal knee joint and knee joint with mild to severe OA. Furthermore, all measures showed a linear increase as OA severity worsened. This suggests that there is a relationship between OA and BMD in the feline knee joint, which can be preliminarily confirmed by this study.

**Abstract:**

Osteoarthritis (OA), including knee joint OA, is a common chronic condition in cats. In both cats and humans, knee joint OA is characterized radiographically by the presence of osteophytes, enthesiophytes, subchondral sclerosis, and joint space narrowing. However, only in humans have these radiographic signs been reported to increase bone mineral density (BMD). Therefore, this study aims to quantify the volumetric (vBMD) and relative (rBMD) BMD measures of the feline knee joint and compare BMD measures between various severities of OA to test the hypothesized OA–BMD relationship in the knee joint in cats. The 46 feline knee joints were imaged using computed tomography (CT) and conventional radiography supported by the computed digital absorptiometry (CDA) method to obtain vBMD and rBMD, respectively. Both BMD measures were assessed in three regions of interest (ROIs): the distal femur (ROI 1), patella (ROI 2), and proximal tibia (ROI 3). In all locations, vBMD and rBMD showed moderate (ROI 2: r = 0.67, *p* < 0.0001) to strong (ROI 1: ρ = 0.96, *p* < 0.0001; ROI 3: r = 0.89, *p* < 0.0001) positive correlations. Due to differences (*p* < 0.0001) in the width of the distal femur (17.9 ± 1.21 mm), patella (8.2 ± 0.82 mm), and proximal tibia (19.3 ± 1.16 mm), the rBMD was corrected (corr rBMD) using the thickness coefficient of 0.46 ± 0.04 for ROI 2 and 1.08 ± 0.03 for ROI 3. Regardless of the quantification method used, BMD measures increased linearly from a normal knee joint to severe OA, with differences in BMD between normal and mild to severe knee joint OA. The OA–BMD relationship in the feline knee joint can be preliminarily confirmed.

## 1. Introduction

Osteoarthritis (OA), also known as degenerative joint disease (DJD), is a common chronic condition in domesticated cats [1,2,3,4,5,6,7]. OA is estimated to affect 91% of the feline population, with the knee joint being involved in 50% of OA-affected felines [5].

In feline knee joint OA, symptoms of pain and joint stiffness significantly impact several important variables, including a joint’s range of motion [8], activity levels [9,10], and quality of life [11,12], particularly among aging [1,7] and overweight [13] populations. In humans, clinical symptoms such as pain, joint stiffness, and impaired mobility have been strongly positively correlated with radiographic signs of knee joint OA [14]. Similarly, in cats, radiographic features of OA have been shown to be correlated with clinical symptoms such as pain, swelling, crepitus, and limitation of range of motion of OA–affected joints [8]. This relationship makes sense when considering the pathogenesis of OA, which involves cartilage degradation, hyperplasia, and hypertrophy, as well as synovial inflammation, subchondral bone sclerosis, and new bone formation. When OA-related joint remodeling involves bone, the signs of OA become identifiable radiologically, which can assist with the diagnosis of OA and the assessment of disease severity and allows us to monitor the progression of disease based on radiographic imaging [1,2,3,5,13,15]. Radiographic signs of feline knee joint OA include the presence of narrow and irregular joint space, periosteal proliferation, osteophytes and/or enthesiophytes, subchondral bone cyst and/or sclerosis, and intra-articular mineralization [1,2,3,4,5,8,13,16,17,18,19]. Thus far, OA has been defined in terms of clinical symptoms, radiographic signs, or a combination of these features [20]. Some studies have also explored the relationship between OA and bone mineral density (BMD) in humans [21,22,23,24]. In these studies, OA was defined solely by radiographic criteria rather than by a combination of radiographic and clinical criteria or clinical criteria alone.

In humans, the presence of osteophytes, enthesiophytes, subchondral sclerosis, and joint space narrowing has been shown to correlate with increased BMD [22,23,24]. Additionally, strong positive correlations have been observed between knee joint OA severity and increased BMD measured in the femoral neck [22,23,24,25,26,27,28] and lumbar spine [25,26,27,28]. Longitudinal prospective studies have also shown an association between increased BMD and an increased risk of developing knee joint OA and its radiographic signs [22,23,24,27,28]. However, no studies to date have investigated the OA–BMD relationship in the feline knee joint. Understanding this relationship may further illuminate the role of bone remodeling in OA pathogenesis, which could have significant therapeutic implications in both human [21] and veterinary medicine. Therefore, this preliminary research on feline specimens is needed.

To date, BMD has been investigated in healthy cats [29,30,31,32,33] and in cats with various diseases [34,35,36,37] but never in those with knee joint OA. The effects of BMD were evaluated in cases of feline mucopolysaccharidosis [35] and secondary hyperparathyroidism [36] using dual-energy X-ray absorptiometry (DXA/DEXA), as well as in cases of chronic dietary acidification [34], secondary hyperparathyroidism [36], and osteogenesis imperfecta [37] using quantitative computed tomography (QCT). The DEXA method was developed to measure bone mineral content in human osteoporosis [38] and has become the ‘gold standard’ for BMD assessment in humans [21]. However, its application in cats, although possible [29,33,35,36], presents clinical limitations. Firstly, the DEXA method requires the use of an expensive DEXA scanner, which is not available in the majority of veterinary clinics, including university clinics [32,33,35,36]. Secondly, DEXA scans measure areal BMD, which is affected by bone size [39,40]. Since increases in bone size in individuals with OA have been observed in both humans [39,40,41] and cats [5,8,10], differences in bone size could confound the OA–BMD relationship [42], leading to an overestimation of BMD proportional to the size of the bone affected [43]. Measuring the volumetric BMD (vBMD) using QCT has been suggested as a method to avoid this problem [21]. Unfortunately, both DEXA and QCT methods require general anesthesia [31,32,33,34,35,36], which significantly hinders the recruitment of feline volunteers for research [31] and is the third limitation of the use of the DEXA method. 

Therefore, in this study, computed digital absorptiometry (CDA) has been introduced as an indirect method of BMD assessment using conventional radiography, which is the primary method for the initial diagnosis of feline knee joint OA and does not require general anesthesia [2,5,8,13]. The CDA method uses an aluminum density standard, which serves as a reference for the measurement of X-ray beam attenuation [44,45,46,47,48,49,50,51], and returns the X-ray detector shading for the examined body part in grayscale [44,45,46,47,48,49,50] or with color annotation [51]. It is then compared with the detector shading for the aluminum density standard, allowing for the estimation of the relative BMD (rBMD). The CDA method has been calibrated [44,45] and applied [46,47,48,49,50,51] in veterinary medicine. It has been used to evaluate skeletal development in young horses [46], monitor risk periods during training [46], predict bone stress injuries [47] and fractures [48], as well as assess the severity of bone [49,51] and dental [50] diseases. However, the CDA method has not yet been used in cat imaging.

Therefore, the aim of this preliminary study was to quantify the BMD of the feline knee joint using CT and CDA methods and compare them between types of studied measures and with various severities of OA to test the hypothesized OA–BMD relationship in the knee joint in cats.

## 2. Materials and Methods

### 2.1. Study Design

This prospective observational study enrolled 23 euthanized cats. The reasons behind the euthanasia of the cats were unrelated to the current study. The cats were presented to the clinic with spine injuries, with no knee joint injuries observed in any of them. The owners reported no history of hyperparathyroidism, osteopenia, osteopetrosis, osteogenesis imperfecta, or mucopolysaccharidosis.

**Figure 1:**
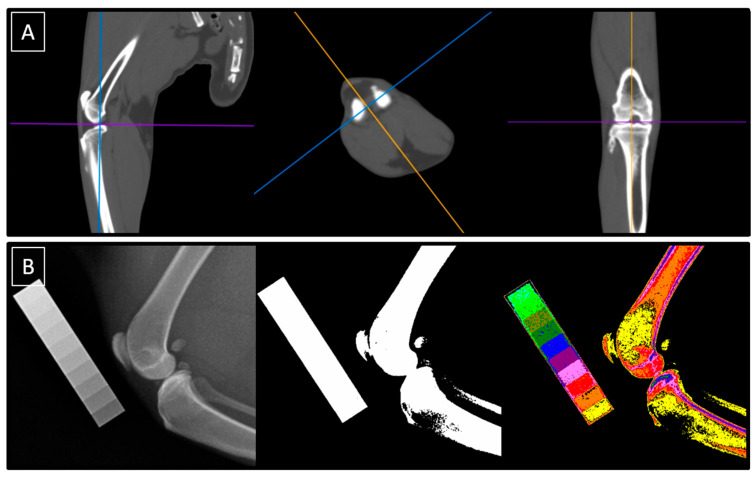
The imaging protocol was conducted using both computed tomography (CT) (**A**) and conventional radiography supported by computed digital absorptiometry (CDA) (**B**). Tomograms were evaluated in the sagittal, transverse, and coronal planes (**A**), while radiographs were decomposed using the CDA method into white-annotated and color-annotated images (**B**).

All cats underwent the same protocol for diagnostic imaging of the knee joints. Both the right and left knee joints were imaged in each cat, for a total of 46 knee joints imaged. The imaging protocol included CT and conventional radiography supported by the CDA methods, as shown in Figure 1. Tomograms were qualitatively evaluated in the sagittal, transverse, and coronal planes, while radiographs were decomposed using the CDA method into the white-annotated images representing bone and color-annotated images represented different degrees of X-ray beam attenuation scaled using the aluminum density standard. The CT scans were performed at the Large Animal Clinic, while conventional radiography was performed at the Small Animal Clinic, both at the Institute of Veterinary Medicine at the Warsaw University of Life Sciences.

### 2.2. Image Collection

#### 2.2.1. Image Collection Using Computed Tomography

The cats were imaged using a 64-slice CT scanner (Revolution CT, GE Healthcare, Chicago, IL, USA). The imaging was performed using a helical scan type with a current of 275 mA, a voltage of 120 kV, a gantry rotation of 0.08/s/HE+, a table travel of 39.4 mm/rotation, a pitch of 0.984:1, and a slice thickness of 0.625 mm. The cats were positioned in sternal recumbency with their pelvic limbs straightened backward. The scan length was adjusted between the cranial aspect of the pelvis and the caudal aspect of the tarsal joint to cover the entire femur, knee joint, and tibia. Therefore, the number of slices was tailored to each cat’s size and differed between scans. Imaging was obtained at a 16-bit quality resolution, and gray levels were displayed in Hounsfield units (HUs). The obtained images were saved in DICOM format using the AW workstation (GE Healthcare, Chicago, IL, USA) and Volume Share software version 7 (GE Healthcare, Chicago, IL, USA).

#### 2.2.2. Image Collection Using Computed Digital Absorptiometry

The cats were then imaged using an X-ray CPI Indico IQ system (Communications & Power Industries Canada Inc., Georgetown, DC, Canada). Mediolateral radiographs of the knee joint were obtained using a current of 1.2 mA, voltage of 60 kV, focus–table distance of 90 cm, and positioning the X-ray beam at the midpoint of the knee joint. The cats were positioned in lateral recumbency with the knee joint freely flexed. The radiograph size was adjusted to the cat’s size so that the desired structures (distal femur, knee joint, and proximal tibia) were imaged. The aluminum density standard (characterized in detail by Górski et al. [50]; mass: 9.39 g; density: 2.65 g/cm^3^; aluminum mass content: 95.20–98.88%; and aluminum atom content: 92.71–98.92%) was positioned next to the knee joint, parallel to the long axis of the patella. In each image, the knee joint and the aluminum density standard were visible. The aluminum density standard served as a reference for image decomposition using a previously described protocol [49,50,51]. The obtained images were saved in DICOM format using the X-ray CPI Indico IQ system (Communications & Power Industries Canada Inc., Georgetown, DC, Canada) and Ubuntu software (Canonical Ltd. Ubuntu Foundation, Isle of Man, Great Britain).

### 2.3. Image Classification

**Figure 2:**
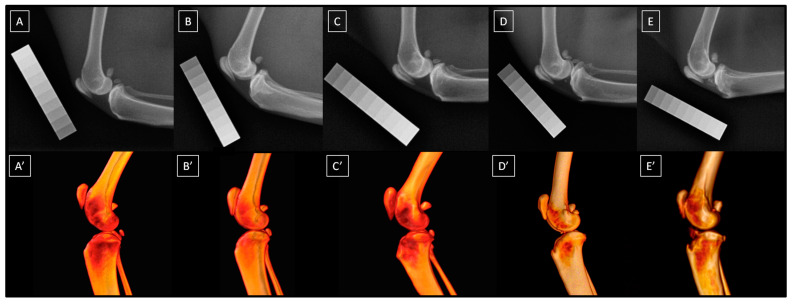
The radiographs (**A**–**E**) and volume renderings (**A’**–**E’**) of feline knee joints are classified as follows: a normal joint (**A**,**A’**), minor osteoarthritis (OA) (**B**,**B’**), mild OA (**C**,**C’**), moderate OA (**D**,**D’**), and severe OA (**E**,**E’**).

The presence and severity of knee joint OA were assessed radiographically using a 5-point scale (0–4) proposed by Lascelles et al. [5] and described in detail by Bonecka et al. [13]. In this scale, a normal knee joint is assessed as 0, minor OA as 1, mild OA as 2, moderate OA as 3, and severe OA as 4. The scale considers the assessment of joint space (width and shape), cortical bone surface (osteophytes and enthesiophytes), subchondral bone (cyst and sclerosis), periosteal proliferation, and intra-articular mineralization. A detailed description of the radiographic signs for each grade of this 5-point scale can be found in Bonecka et al. [13]. Example radiographs and volume renderings from CT imaging of the same cats for each grade of this 5-point scale are presented in Figure 2. Cats were imaged until at least five knee joints were classified as belonging to each of the five OA-related groups. Once this limit was met, the recruitment of new individuals ceased.

### 2.4. Data Quantification

#### 2.4.1. Quantification of Computed Tomography Images

**Figure 3:**
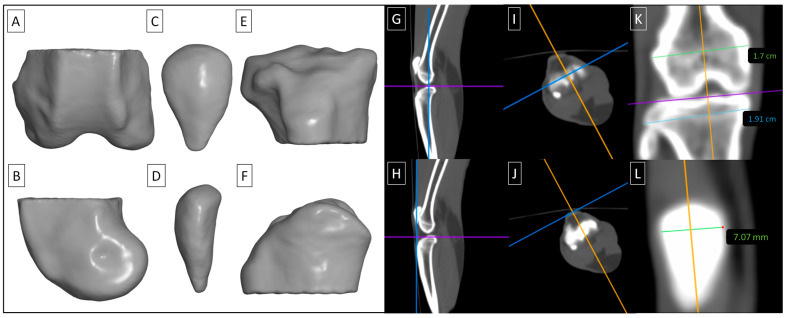
Three regions of interest (ROIs) were segmented in each knee joint, including the distal epiphysis of the femur (ROI 1) (**A**,**B**), the patella (ROI 2) (**C**,**D**), and the proximal epiphysis of the tibia (ROI 3) (**E**,**F**). ROIs are displayed in a dorsocaudal view (**A**,**C**,**E**) and mediolateral view (**B**,**D**,**F**). The tomograms were positioned in the sagittal (**G**,**H**), transverse (**I**,**J**), and coronal (**K**,**L**) planes for measuring the width of the distal femur (**G**,**I**,**K**), patella (**H**,**J**,**L**), and proximal tibia (**G**,**I**,**K**).

Three regions of interest (ROIs) were semi-automatically segmented on each limb following ranges suggested by Boyd et al. [45]. ROI 1 represented the distal epiphysis of the femur (Figure 3A,B), ROI 2 represented the patella (Figure 3C,D), and ROI 3 represented the proximal epiphysis of the tibia (Figure 3E,F). Segmentation was conducted using Materialises Interactive Medical Image Control System (MIMICS) software version 14.0 (Materialise HQ, Leuven, Belgium). The tomograms in DICOM format were imported into MIMICS software, and gray level mapping was set between −1023 HU and 3056 HU. Tissues with a density ≥450 HU [45] were semi-automatically annotated and then corrected manually to separate the representative ROIs. For each segmented ROI, the vBMD values were extracted in HU.

Then, the tomograms were displayed using a bone window set at a level of +350 and a width of 2000 using Osirix MD software version 12.0 (Pixmeo SARL, Bernex, Switzerland). This setting was used to measure the width of the desired anatomical structures, with the knee joints always positioned in the same manner in the sagittal (Figure 3G,H), transverse (Figure 3I,J), and coronal (Figure 3K,L) planes. All measurements were carried out on images in the coronal plane. The width of the distal epiphysis of the femur (distal femur) was measured as the longest distance between the lateral and medial epicondyles, parallel to the joint space (Figure 3G,I,K). The width of the patella was measured in the widest point (Figure 3H,J,L). The width of the proximal epiphysis of the tibia (proximal tibia) was measured as the longest distance between the lateral and medial condyles, parallel to the joint space (Figure 3G,I,K).

#### 2.4.2. Quantification of Computed Digital Absorptiometry Images

Radiographs were decomposed using MIMICS software. In each image, the minimum (Min), maximum (Max), and mean (Mean) standard attenuation (SA) values of the X-ray beam were determined for each of the ten steps (S1–S10) of the aluminum density standard (Table 1). The SA was recorded in HU. The respective ranges were masked with an assigned color on the entire radiograph, following the previously described protocol [51]. The entire range, from the Min SA for S1 and the Max SA for S10, was masked in white (HEX #FFFFFF). As a result, ten color-annotated images and one white-annotated image were produced for each radiograph. The white-annotated images represented the entire bone annotation. The decomposed images were resized to 621 × 483 pixels and saved as separate BMP files.

**Figure 4:**
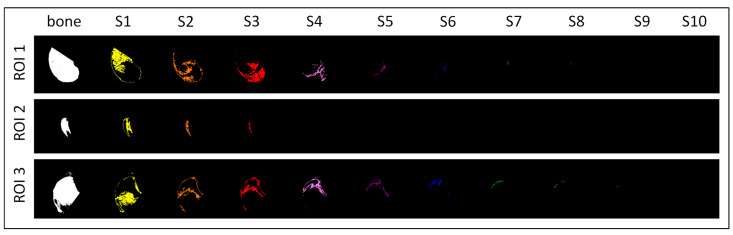
Three regions of interest (ROIs) were segmented in each knee joint, including the distal epiphysis of the femur (ROI 1), the patella (ROI 2), and the proximal epiphysis of the tibia (ROI 3). For each knee joint, ROIs were segmented on one white-annotated image (representing the entire bone) and ten color-annotated images represented ten steps (S1–S10) of the aluminum density standard.

Then, the decomposed images were manually segmented using ROIs corresponding to the tomogram ROIs (see Section 2.4.1). Segmentation was conducted using Paint.NET software version 4.3.2. The decomposed images in BMP files were imported into Paint.NET software. Cutting lines in the HEX color #000000 were set at the same levels as those for the tomograms. The entire area outside the cutting lines was manually masked using the HEX color #000000. This procedure was repeated three times on each raw decomposed image, ensuring that each ROI was saved as a separate BMP file (Figure 4).

For each segmented ROI, the color pixels were quantified using the previously described color pixel-counting protocol [51]. The number of pixels of each color was returned from both color-annotated and white-annotated images. This procedure was repeated for each ROI separately. Then, mean SA values (see Table 1) were used for the rBMD calculation according to the following Equation (1).

(1) $$rBMD=\frac{\sum_{i=1}^{10}\left(MeanSA(S_{i})\cdot s_{i}\right)}{N_{ROI}}$$

where $Mean\;SA(S_{i})$ is the mean standard attenuation (Mean SA) of the X-ray beam for each of the S1–S10 steps, $s_{i}$ is the number of color pixels for each color in the ROI, and $N_{ROI}$ is the number of white pixels in the ROI. The rBMD values were expressed in HU.

### 2.5. Statistical Analyses

The data set included the vBMD, rBMD, and bone width data series for each ROI, respectively. Each data series contained 46 realizations, with 1 realization corresponding to one knee joint. To compare the BMD of three-dimensional (vBMD) and two-dimensional (rBMD) images, the rBMD correction was introduced by dividing by the thickness coefficients (*a*1, *a*2, and *a*3). The thickness coefficients were calculated as the relative width of the assessed anatomical structure, with the width of the distal femur taken as the reference. Thus, for the distal femur (ROI 1), *a*1 was 1; for the patella (ROI 2), *a*2 = (width of the patella)/(width of the distal femur); and for the proximal tibia (ROI 3), *a*3 = (width of the proximal tibia)/(width of the distal femur). The corrected rBMD (corr rBMD) was calculated for ROI 2 and ROI 3 as follows: corr rBMD = rBMD/*a*, for *a*2 and *a*3, respectively. Consequently, additional data series (corr rBMD) were created for ROI 2 and ROI 3.

The normality of the data series was tested using the Kolmogorov–Smirnov normality test. Since not all data series followed a normal distribution, the data were presented in plots using medians and ranges (lower and upper quartiles, as well as minimum and maximum values). Bone width data series were compared between assessed anatomical structure as paired data using the Friedman test followed by the Dunn’s multiple comparisons test. BMD data series were compared between BMD measures (vBMD vs. rBMD and vBMD vs. corr rBMD) within individual locations (ROI 1, ROI 2, and ROI 3) using the paired *t*-test for a pair of Gaussian data series or the Wilcoxon matched-pairs signed-rank test for at least one non-Gaussian data series. The Pearson correlation coefficient (r) was calculated for a pair of Gaussian data series, while Spearman’s rank correlation coefficient (ρ) was calculated for at least one non-Gaussian data series. Correlations were considered significant when *p* < 0.05.

Subsequently, the data series were divided into five OA-related groups based on the image classification (see Section 2.3). The normality of the new data series was tested using the Kolmogorov–Smirnov normality test. Data series were compared between OA-related groups (0–4 OA severity) within individual locations (ROI 1, ROI 2, ROI 3) for each BMD (vBMD, rBMD, corr rBMD) separately. One-way ANOVA was used for Gaussian data series, and the Kruskal–Wallis test was used when at least one data series was non-Gaussian. When *p* < 0.05, post hoc multiple comparison tests were used: Holm–Sidak’s for Gaussian data series and Dunn’s test for non-Gaussian data series. 

For BMD measures (vBMD vs. rBMD and vBMD vs. corr rBMD), linear regressions were calculated in each ROI separately. The regression equations were displayed on regression plots, and the differences in linearity were calculated. All slopes were significantly non-zero (*p* < 0.0001); therefore, the slopes were compared. When the slopes did not differ significantly (*p* > 0.05), a single slope was calculated, and the intercepts were compared.

Statistical analysis was performed using GraphPad Prism version 6 (GraphPad Software Inc., San Diego, CA, USA). Statistical significance was set at *p* < 0.05.

## 3. Results

### 3.1. Descriptive Data

This study enrolled 14 male and 9 female European breed cats, aged between 7 and 15 years. Of the 46 knee joints included in this study, 6 joints were classified as normal (OA grade 0), 17 as minor OA (OA grade 1), 12 as mild OA (OA grade 2), 6 as moderate OA (OA grade 3), and 5 as severe OA (OA grade 4). The limiting group for meeting the sample size target was the OA grade 4 group. After filling this group with five joints, the recruitment of individuals ceased.

The width of the assessed anatomical structures without grouping by OA severity is summarized in Table 2. For the entire data set, the mean width ± standard deviation (SD) of the distal femur was 17.9 ± 1.21 mm, the patella was 8.2 ± 0.82 mm, and the proximal tibia was 19.3 ± 1.16 mm. The bone width differed between each individual location, with the lowest being in the patella, increased in the distal femur, and highest in the proximal tibia. Thickness coefficients were calculated individually for each knee joint. For the entire data set, the mean ± SD of *a*2 was 0.46 ± 0.04 while *a*3 was 1.08 ± 0.03, with *a*1 always equal to 1.

### 3.2. BMD of the Distal Femur, Patella, and Proximal Tibia

**Figure 5:**
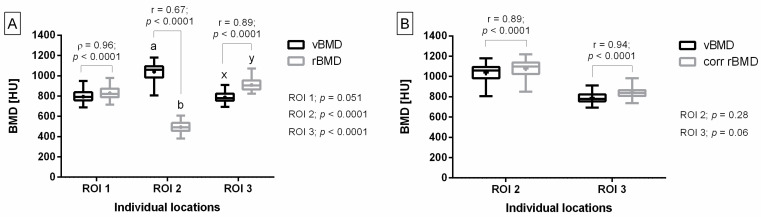
Bone mineral density (BMD) measures, including volume BMD (vBMD), relative BMD (rBMD), and corrected rBMD (corr rBMD), compared at three locations in the feline knee joint before (**A**) and after (**B**) thickness-related correction. BMD measures were compared for the following regions of interest (ROIs) separately: ROI 1 represents distal femur, ROI 2 represents patella, and ROI 3 represents proximal tibia. Data on box plots are represented by lower quartile, median, and upper quartile, whereas whiskers represent minimum and maximum values. Additionally, the mean values are marked by “+”. Lowercase letters (a, b; x, y) indicate differences between BMD measures where *p* < 0.05. *p* values were displayed for each ROI separately. Spearman’s rank correlation coefficient (ρ) (for ROI 1 on subfigure (**A**)) and the Pearson correlation coefficient (r) (for ROIs 2–3 on subfigures (**A**,**B**)) were displayed for each data pair. Correlations are significant when *p* < 0.05.

In ROI 1, a strong positive correlation was found between vBMD and rBMD, with no difference between the values of these two measures. However, in ROI 2, rBMD was decreased compared to vBMD, while in ROI 3, rBMD was increased compared to vBMD. In both ROIs, positive correlations—moderate in ROI 2 and strong in ROI 3—were found between vBMD and rBMD (Figure 5A).

After correction of rBMD, no differences were found between vBMD and corr rBMD in both ROI 2 and 3. In both ROIs, strong positive correlations were found between vBMD and corr rBMD (Figure 5B).

### 3.3. OA-BMD Relationship in the Distal Femur, Patella, and Proximal Tibia

**Figure 6:**
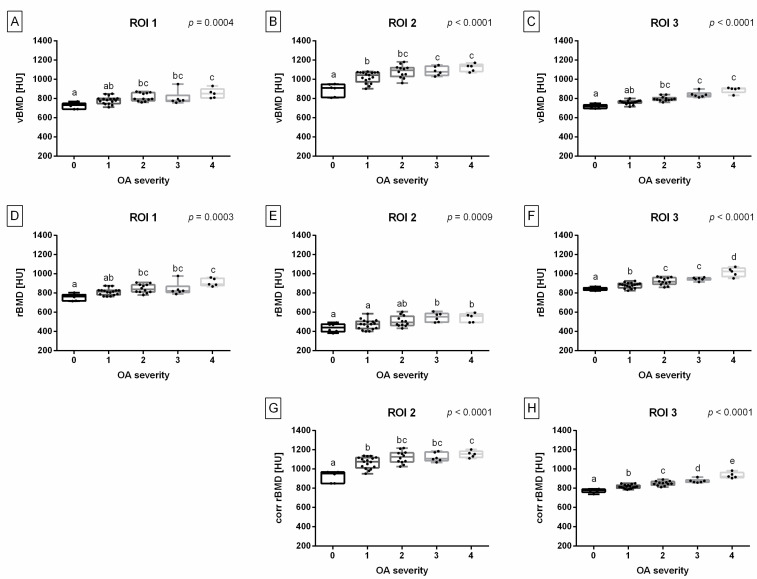
Bone mineral density (BMDs) measures, including volume BMD (vBMD) (**A**–**C**), relative BMD (rBMD) (**D**–**F**), and corrected rBMD (corr rBMD) (**G**,**H**), compared between groups of osteoarthritis (OA) severity (grades 0–4). BMD measures were compared for the following regions of interest (ROIs) separately: ROI 1 represents distal femur (**A**,**D**), ROI 2 represents patella (**B**,**E**,**G**), and ROI 3 represents proximal tibia (**C**,**F**,**H**). Data points on box plots are represented by lower quartile, median, and upper quartile, whereas whiskers represent minimum and maximum values. Additionally, each realization is marked by a point. Lowercase letters (a–e) indicate differences between OA-related groups when *p* < 0.05. *p* values were displayed for each ROI separately.

In all studied ROIs, BMD measures increased with OA grades; however, the magnitude and significance of this increase varied between individual locations and BMD measures (Figure 6).

In ROI 1, vBMD was increased in OA grades 2–4 compared to OA grade 0; and increased in OA grade 4 compared to OA grade 1 (Figure 6A). In ROI 2, vBMD was the lowest in OA grade 0, increased in OA grade 1, and the highest in OA grades 3–4 (Figure 6B). In ROI 3, vBMD was increased in OA grades 2–4 compared to OA grade 0; and increased in OA grades 3–4 compared to OA grade 1 (Figure 6C).

In ROI 1, rBMD was increased in OA grades 2–4 compared to OA grade 0, and increased in OA grade 4 compared to OA grade 2 (Figure 6D). In ROI 2, rBMD was increased in OA grades 3–4 compared to OA grades 0–1 (Figure 6E). In ROI 3, rBMD was the lowest in OA grade 0, increased in OA grade 1, increased again in OA grades 2–3, and the highest in OA grade 4 (Figure 6F). In ROI 2, corr rBMD was increased in OA grades 1–4 compared to OA grade 0, and increased in OA grade 4 compared to OA grade 1 (Figure 6G). In ROI 3, corr rBMD was the lowest in OA grade 0 and then gradually increased to the highest value in OA grade 4, with significant differences between each grade (Figure 6H).

**Figure 7:**
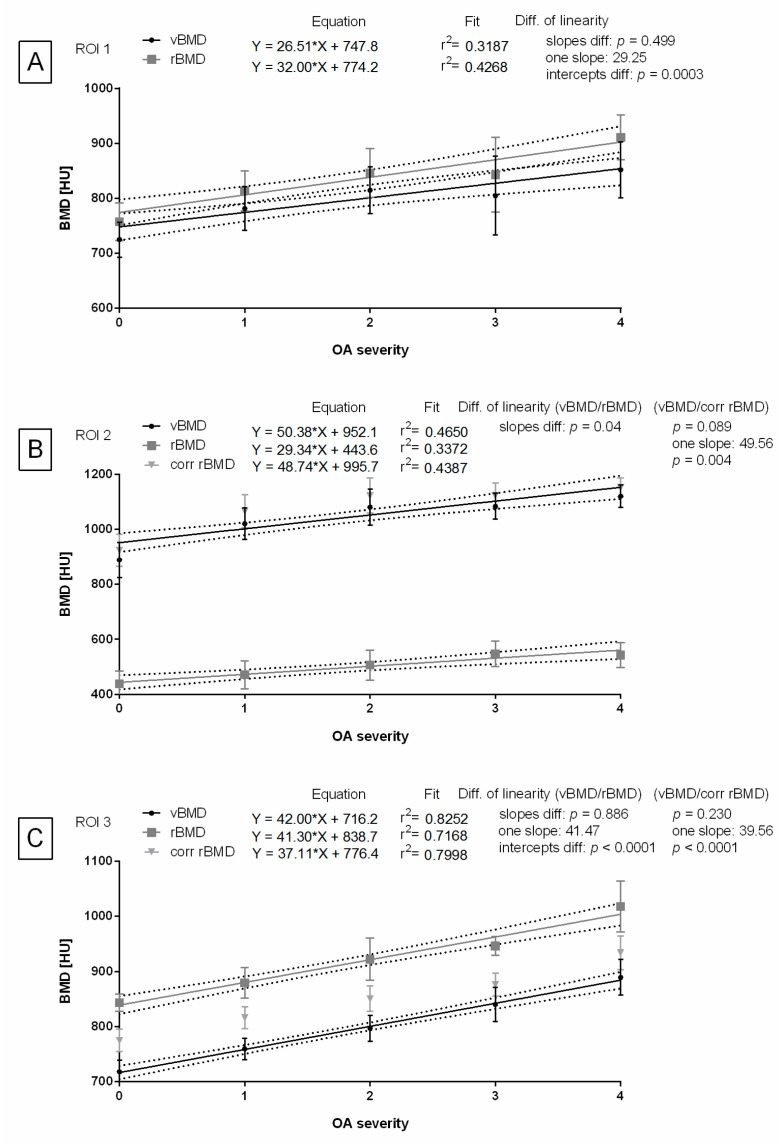
Linearity of the osteoarthritis-bone mineral density (OA–BMD) relationship. The regression lines reflecting BMD measures with increasing OA severity (grades 0–4) are displayed for volume BMD (vBMD), relative BMD (rBMD), and corrected rBMD (corr rBMD). Linearity was compared for the following regions of interest (ROIs) separately: ROI 1 represents distal femur (**A**), ROI 2 represents patella (**B**), and ROI 3 represents proximal tibia (**C**). Linearity was considered similar when *p* < 0.05. When similarity between slopes was confirmed (*p* < 0.05), a single slope measurement was calculated, and the intercepts were compared.

In ROI 1, the slopes of the linear regression equations for vBMD and rBMD were not significantly different, allowing for the calculation of one slope (one slope: 29.25) and comparison of the intercepts. The difference between the intercepts for vBMD and rBMD was significant (Figure 7A).

In ROI 2, the slopes of the linear regression equations for vBMD and rBMD were significantly different. However, the slopes for vBMD and corr rBMD were not significantly different. Therefore, for the latter data pair, one slope was calculated (one slope: 49.56) and the intercepts were compared. The difference between the intercepts for vBMD and corr rBMD was significant (Figure 7B).

In ROI 3, the slopes of the linear regression equations for both data pairs, vBMD and rBMD as well as vBMD and corr rBMD, were not significantly different. Thus, a shared slope was calculated for vBMD and rBMD (one slope: 41.47), and another shared slope was calculated for vBMD and corr rBMD (one slope: 39.56). The respective intercepts were then compared. For both data pairs, the differences between the intercepts were significant (Figure 7C).

## 4. Discussion

The obtained results allow us to preliminarily accept the hypothesis that, like in humans [22,23,24,25,26,27,28], BMD is associated with knee joint OA in cats. Moreover, BMD differed between OA grades, and these differences were best illustrated in the proximal tibia. This finding suggests that this individual location exhibits the most significant radiological signs of bone density.

In humans, the presence of osteophytes and/or enthesiophytes and subchondral bone sclerosis can increase BMD [21,24]. However, these studies did not precisely determine the relationship between specific radiological signs and BMD, which is a limitation of these studies. Clarke et al. [2] showed that in cats, the main radiological signs of knee joint OA are the presence of enthesiophytes at the insertion of the straight patellar ligament onto the tibial tuberosity and intra-articular mineralization. In this study, these ‘bone-forming’ changes were observed in ROI 3 when segmented and could potentially contribute to an increase in BMD. In human studies where specific radiographic signs of OA were quantified, increased BMD predominantly co-occurred with osteophytosis [22,23], while the co-occurrence of BMD with joint space narrowing was rarely demonstrated [24]. It has been observed that humans with higher BMD exhibit a ‘bone-forming’ tendency [21], suggesting that further research targeting the causal factors that mediate the relationship between OA and BMD is needed. Both higher BMD [22,23,24,27] and a ‘bone-forming’ tendency [21] in humans are associated with radiographic signs of knee joint OA. Therefore, in cats, further research is needed to identify specific radiological signs of knee joint OA, quantify them separately, and correlate their appearance with BMD.

Interestingly, in humans with knee joint OA, variations in BMD have been noted in different subchondral bone regions [52,53]. For example, the subchondral bone of the arthritic joint may be thickened compared to a normal joint, and in some cases, the trabecular bone underlying the thickened subchondral bone may exhibit osteopenic radiological signs. This variation is termed ‘stress-shielding’ [54]. In humans, the architecture and strength of the subchondral bone and adjacent trabecular bone have been shown to be sensitive to acute knee joint injuries [6,7]. Similarly, in cats, increased BMD of the subchondral bone has been suggested as an adaptation to post-traumatic knee joint OA [55].

In humans, joint instability and changes in loading characteristics following injury or experimental intervention are accepted as risk factors for OA development and progression [56]. However, in cats, the mechanics of a joint that is predisposed to OA seem to be slightly different. An acute knee joint injury results in significant changes in joint loading, whereas progressive osteoarthritic changes have not been shown to cause a general reduction in mechanical loading [9]. Therefore, monitoring cats after trauma and those with overloaded knee joints may be crucial for the early detection of knee joint OA while monitoring older cats [5,7] and cats with diagnosed knee joint OA [5,57,58,59] may be important for planning or modifying therapy and rehabilitation.

It is worth noting that age is currently the only confirmed feline risk factor for OA prevalence [5,7] and exacerbation of OA clinical symptoms [8]. Clinical symptoms, particularly signs of pain, did not correlate with high OA severity, as defined radiographically [8]. The concordance between radiographic signs and clinical symptoms of knee joint OA is also limited in humans [60]. However, the degree of BMD elevation was similar in humans with both radiographic and clinical OA compared to those with radiographic OA but no clinical symptoms [21]. In both humans [21,61] and cats [7,8], symptomatic knee joint OA is less common than radiographic knee joint OA. Although in humans, pain signs are mainly self-reported [62] and clinical symptoms are easier to assess than in cats [8], only a few human studies describe the OA-BMD relationship at the level of clinical symptoms [21]. It should be noted that at this stage of the study, the results of the clinical examination were not considered because the BMD measurements were performed on euthanized cats. While this approach is beneficial for preliminary studies, as it bypasses the need for ethics committee approval, it also introduces a significant clinical limitation. The use of euthanized cats precluded the assessment of key clinical symptoms of OA in cats, such as knee joint pain, swelling, crepitus, and limited range of motion [8]. Additionally, feline diseases that may systemically affect BMD—such as primary and secondary hyperparathyroidism [34,63], osteopenia, osteopetrosis [64], osteogenesis imperfecta [37], and mucopolysaccharidosis [35,65]—were excluded only based on the cats’ history. Therefore, further research is strongly needed to explore the relationship between clinical knee joint OA and BMD, including also the use of laboratory tests to exclude the aforementioned systemic diseases as part of the criteria for future prospective clinical trials.

The directions for further research and perspectives highlighted here require imaging a large population of cats. This study was conducted on euthanized cats, and although cats did not require general anesthesia, recruiting a sufficiently large group of cats was still challenging. Meeting the target sample size in the severe OA group was particularly time-consuming. As a result, there was an uneven distribution of sample sizes between OA severity groups, which is an additional limitation of this study.

The usage of euthanized cats unfortunately precludes their clinical evaluation. Therefore, further feline studies require imaging of clinical patients, the recruitment of which is very limited due to the need for general anesthesia [31,32,33,34,35,36]. In this study, in addition to CT quantification, an indirect method was used that does not require general anesthesia. Both methods allowed for the quantification of BMD in the feline knee joint, demonstrating moderate to strong positive correlations between vBMD and rBMD. The results obtained via the CDA method differed between each OA-dependent group and more strongly correlated with vBMD after considering the size of the imaged structure. Thus, it is advisable to implement the thickness coefficient when interpreting BMD data. Integrating the CDA method into standard feline clinical practice may be beneficial for providing more radiographic data for further research.

### Further Directions

The existence of an association between BMD and knee joint OA is generally accepted in the case of humans [20,21,22,23,24,25,26,27,28], and this study provides support for the same relation in cats. This is evidenced, regardless of the method used for BMD quantification, by the linear, gradual increase in BMD with OA severity and by the differences in BMD between normal and mild to severe knee joint OA.

However, the mechanisms underlying these findings remain unclear and require further research. In humans, factors such as thinning of the articular cartilage [52], altered stiffness of subchondral bone [66], and changes in osteoblast differentiation [67]—potentially mediated by transforming growth factor-β (TGF-β) and/or the Wnt signaling pathways [68]—are suggested to be involved in the BMD-related pathogenesis of OA. No similar studies have been conducted in cats, although similar comparisons have been made in veterinary medicine regarding the potential for studying human and animal biomarkers of OA [69]. Therefore, measuring the concentration of particular markers important for OA pathogenesis in synovial fluid [70,71] and conducting genomic studies on OA signaling pathways [72,73] in tissues collected from the knee are expected directions for the advancement of feline knee OA research.

Before these steps are taken, it is necessary to conduct reference tests using the DEXA method [29,33,35,36] to confirm the indirect relationships shown in this study and to relate the measurements to the cat’s clinical symptoms [7,8,10] and individual radiological signs in the knee [13]. We believe that continued research in these areas will contribute to a better understanding of the pathogenesis of feline knee joint OA and facilitate the development of pharmacotherapies targeting bone remodeling to treat OA, particularly with the use of disease-modifying OA drugs (DMOADs) [74]. This further direction in feline research is especially important, considering that the primary treatment approach for feline knee joint OA is symptom-reducing therapy [5,13,57,58,59].

## 5. Conclusions

Both methods allowed for the quantification of BMD in the feline knee joint, showing moderate to strong positive correlations between the types of measures studied. However, when using CDA methods for relative BMD quantification, it is advisable to consider the thickness coefficient of the assessed anatomical structures. Regardless of the quantification method used, the OA–BMD relationship in the feline knee joint can be preliminarily confirmed by the linear, gradual increase in BMD with OA grades, as well as by the differences in BMD between normal and mild to severe knee joint OA.

## Figures and Tables

**Table 1:** The colors and HEX codes assigned to the ten steps (S1–S10) of the aluminum density standard, along with the minimum (Min), maximum (Max), and mean (Mean) standard attenuation (SA) values of the X-ray beam.

Decomposition	S1	S2	S3	S4	S5	S6	S7	S8	S9	S10
Color	Yellow	Orange	Red	Light purple	Dark purple	Dark blue	Dark green	Navy green	Light green	Light blue
HEX code	#FFFF00	#E08000	#FF0000	#E080C0	#800080	#0000FF	#008000	#808000	#00FF00	#A6CAF0
Min SA [HU]	−330.0	63.3	465.0	837.8	1125.6	1291.9	1554.4	1741.1	1965.6	2156.7
Max SA [HU]	919.4	1191.7	1446.7	1659.4	1821.1	2018.9	2181.1	2268.3	2381.1	2539.4
Mean SA [HU]	294.7	627.5	955.9	1248.6	1473.4	1655.4	1867.8	2004.7	2173.4	2348.1

**Table 2:** The thickness coefficients (*a*1, *a*2, and *a*3) calculated based on the width of the distal femur, patella, and proximal tibia for studied regions of interest (ROIs), representing the distal femur (ROI 1), patella (ROI 2), and proximal tibia (ROI 3). The values are reported as mean ± standard deviation (SD). Lowercase letters (a–c) indicate differences in the width between individual locations when *p* < 0.05.

Width (mm).				Thickness Coefficient		
distal femur	patella	proximal tibia	*p*	ROI 1 (*a*1)	ROI 2 (*a*2)	ROI 3 (*a*3)
17.9 ± 1.21 ^a^	8.2 ± 0.82 ^b^	19.3 ± 1.16 ^c^	<0.0001	1	0.46 ± 0.04	1.08 ± 0.03

## Data Availability

The data presented in this study are available upon request from the corresponding author.
